# Prognonstic impact of renin-angiotensin system blockade in localised upper-tract urothelial carcinoma

**DOI:** 10.1038/bjc.2011.565

**Published:** 2011-12-20

**Authors:** N Tanaka, A Miyajima, E Kikuchi, K Matsumoto, M Hagiwara, H Ide, T Kosaka, T Masuda, S Nakamura, M Oya

**Affiliations:** 1 Department of Urology, Keio University School of Medicine, 35 Shinanomachi, Shinjuku-ku, Tokyo 160-8582, Japan; 2 Department of Urology, Saiseikai Central Hospital, Tokyo, Japan; 3 Department of Urology, Saitama Municipal Hospital, Saitama, Japan

**Keywords:** renin-angiotensin system, angiotensin II-converting enzyme inhibitor, angiotensin II receptor blocker, upper-tract urothelial carcinoma

## Abstract

**Background:**

The potential role of the renin-angiotensin system (RAS) in the promotion of tumour growth has been investigated, and the administration of RAS inhibitors, such as angiotensin-converting enzyme inhibitors (ACEIs) or angiotensin II receptor blockers (ARBs), may improve disease control in malignancy. We investigated the prognostic impact of RAS inhibitors by analysing data from patients with upper-tract urothelial carcinoma (UTUC).

**Methods:**

A total of 279 patients who underwent nephroureterectomy for localised UTUC (pTa-3N0M0) were identified at our three institutions. We retrospectively investigated the prognostic outcomes following nephroureterectomy in patients administered or not administered ACEIs or ARBs.

**Results:**

The median follow-up period was 3.4 years. RAS inhibitors were administered to 48 patients (17.2%). Multivariate analysis showed that the appearance of pathological T3, positive lymphovascular invasion, and no RAS inhibitor administration (*P*=0.027 HR=3.14) were independent risk factors for a decrease in subsequent metastasis-free survival. The 5-year metastasis-free survival rate was 93.0% in patients who administered RAS inhibitors, and 72.8% in their counterparts who did not (*P*=0.008).

**Conclusion:**

The absence of RAS inhibitor administration was an independent risk factor for subsequent tumour metastasis in patients with localised UTUC. We propose RAS inhibitors may be a potent choice as an effective treatment following nephroureterectomy.

Upper-tract urothelial carcinoma (UTUC) is relatively uncommon, accounting for approximately 5% of urothelial malignancies and 10% of all renal tumours (Rouprêt *et al*, 2011; Jemal *et al*, 2011). Surgical resection such as nephroureterectomy with excision of a bladder cuff is the standard treatment for localised UTUC; however, contemporary oncological outcomes still remain poor owing to the high risk of systemic recurrence after operation. Many patients with localised UTUC are stage T3 or greater at the time of surgery (Holmang and Johansson, 2005; Matin *et al*, 2010), and at least 20–25% of these patients may already be considered to have lymph node involvement (Miyake *et al*, 1998; Kondo *et al*, 2007). Even after radical surgery, 5-year disease-specific survivals ranging between 50% and 80% have been demonstrated (Hall *et al*, 1998; Margulis *et al*, 2009; Favaretto *et al*, 2010). Therefore, an important goal of further research is to identify potent therapeutic applications in an attempt to improve disease control following surgery in patients with localised UTUC.

Angiotensin II (Ang II) is a key biological peptide in the renin-angiotensin system (RAS). There are two major subtypes of Ang II receptors; Ang II type 1 receptors (AT1R) and type 2 receptors (AT2R). Concern regarding the potential role of Ang II in angiogenesis and promotion of tumour growth has been growing in the past few decades (Egami *et al*, 2003; Juillerat-Jeanneret *et al*, 2004; Ager *et al*, 2008; George *et al*, 2010), and Lever *et al* reported the first clinical evidence that long-term Ang II blockade might have a protective effect against carcinogenesis (Lever *et al*, 1998). We previously showed that Ang II-AT1R signalling led to potent induction of vascular endothelial growth factor, and RAS inhibition led to an anti-angiogenic effect in urogenital malignancies (Miyajima *et al*, 2002; Kosugi *et al*, 2006; Kosaka *et al*, 2007; Tanaka *et al*, 2010). Using human bladder cancer specimens, we also confirmed that tumours with a high grade and/or high stage would potentially have higher AT1R expression in urothelial cancer cells (Shirotake *et al*, 2011). Thus, we propose that administration of RAS inhibitors, such as angiotensin-converting enzyme inhibitors (ACEIs) or Ang II receptor blockers (ARBs), may improve the control of urothelial malignancies.

In the present study, we retrospectively reviewed the data of 265 patients who underwent nephroureterectomy for localised UTUC. We examined the association between clinicopathological parameters and the use of ACEIs or ARBs, and then analysed outcomes to identify the prognostic impact of RAS inhibitor administration in UTUC.

## Patients and methods

After institutional review board approval, medical records from between 1995 and 2009 archived at Keio University Hospital, Saiseikai Central Hospital, and Saitama Municipal Hospital were retrospectively reviewed. During this period, more than 300 cases underwent nephroureterectomy for UTUC at our three institutions. Five patients with distant metastasis at diagnosis, 14 with pT4 and/or positive lymph node involvement, and four with concomitant muscle-invasive bladder cancer were excluded from study. We identified a total of 284 patients with localised UTUC (pTa-3N0M0) in our study population. After excluding five patients who began to administer RAS inhibitors following nephroureterectomy, the remaining 279 patients were included in the subsequent analyses. The median follow-up for all cohorts was 3.4 years (range, 0.2–15 years).

Open nephroureterectomy was performed in 215 patients (77.1%), whereas the remaining 64 patients (22.9%) underwent laparoscopic nephroureterectomy. Dissection of regional lymph nodes was performed in patients with nodes that were found to be enlarged in a pre-operative evaluation or in those who were suspected for having enlarged nodes at intraoperative inspection. Extended lymphadenectomy was not routinely performed. CDDP-based neoadjuvant and adjuvant chemotherapy regimens were administered to 3 (1.1%) and 43 patients (15.4%), respectively. Post-operative adjuvant radiotherapy regimens were not routinely used.

Patients were assessed by urine cytology and cystoscopy every 3 months for 2 years following nephroureterectomy, every 6 months for the next 3 years, and then every 6–12 months thereafter. CT, and/or MRI, and/or excretory urograms were also performed every 6 months for 5 years and annually thereafter. In Japan, patients have equal access to standard care under the comprehensive Japanese national health-insurance programme, and thus there were most likely no dramatic differences in the clinical course of treatments and follow-up in the study population.

The independent variables used in the present study were patient age, gender, history of cardiovascular disease, chronic kidney disease (CKD), diabetes, hypertension, the use of antihypertensive drugs at nephroureterectomy, status of perioperative chemotherapy, tumour location, tumour length, and pathological features (tumour grade, pathological T stage, appearance of lymphovascular invasion (LVI), and the presence of concomitant carcinoma *in situ* (CIS)) in surgical specimens.

All patients were interviewed by attending physicians at least two times, such as during hospitalisation at the time of diagnosis and at nephroureterectomy, and data concerning their history of disease and medications administered were routinely collected in the study. Moreover, data concerning their medication after surgery was obtained from medical records and interviews by physicians. The use of antihypertensive drugs at nephroureterectomy was reviewed, focusing on the use of RAS inhibitors (ACEIs or ARBs) and other agents (calcium-channel blockers, *β*-blockers, and diuretics). CKD was defined as an eGFR <60 ml/min/1.73 m^2^ according to National Kidney Foundation guidelines (National Kidney Foundation, 2002) and calculated using the four-variable MDRD study equation (Levey *et al*, 1999). Tumour location was divided into two areas: the renal pelvis or ureter based upon the location of the dominant lesion. Tumour size was measured using the maximum diameter of the tumour. With regard to the pathologic disease stage, all neoplasms were classified according to the 2002 TNM staging system. Histologic grades were assigned according to the three-tiered World Health Organization classification. LVI was defined as the presence of tumour cells within an endothelium-lined space without underlying muscular walls. The presence of concomitant CIS was also assessed in every representative section.

The associations between the clinicopathological parameters of a patient and the status of RAS inhibitors were analysed. These associations were validated by the *χ*^2^-test or Mann–Whitney *U*-test. Metastasis-free, disease-specific, and overall survival rates were estimated using the Kaplan–Meier method and were compared by the log-rank test. Survival time was calculated using the data from the operation. Multivariate analysis was performed using the Cox proportional hazards model with stepwise forward selection. Differences among groups were regarded to be significant when *P*<0.05. These analyses were performed with the SPSS version 17.0 statistical software package.

## Results

The mean age of all cohorts was 69 years (range, 36–92 years). Males accounted for 74.2% (207 patients) and females 25.8% (72 patients). Table 1 presents a summary of the clinicopathological parameters for the 279 patients. Of the 279 patients, 128 were diagnosed with hypertension, and a total of 113 patients (40.5%) received medications for hypertension at nephroureterectomy. Of these patients, 48 (17.2%) used ACEIs (*N*=5) or ARBs (*N*=43). Other antihypertensive drugs included calcium-channel blockers (*N*=90), *β*-blockers (*N*=15), or diuretics (*N*=7). The type of ACEI or ARB at surgery is shown in Table 2. Of 48 patients, 45 continued to administer RAS inhibitors with a mean duration of 4.5 years following nephroureterectomy (range, 0.4–13 years). Patient data concerning the duration of RAS inhibitor administration following surgery was not available for three patients.

**Figure 1:**
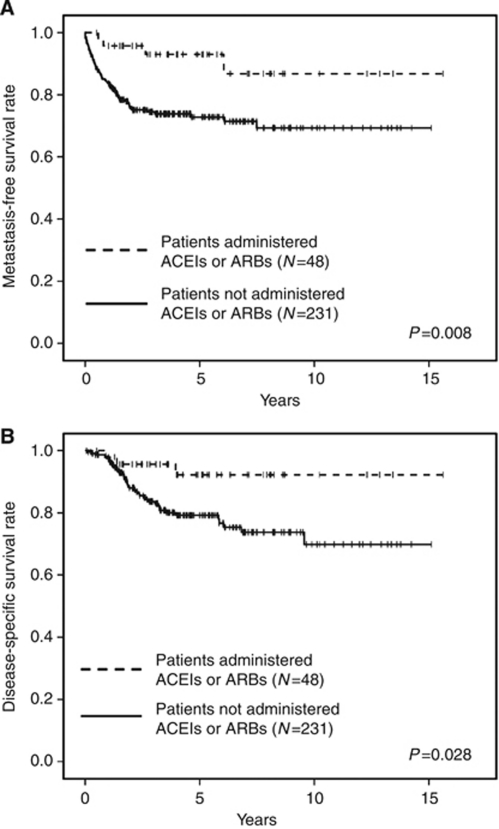
(**A**) Metastasis-free survival rate after nephroureterectomy in patients administered *vs* not administered ACEIs or ARBs. (**B**) Disease-specific survival rate after nephroureterectomy in patients administered *vs* not administered ACEIs or ARBs.

During the follow-up, tumour metastasis was observed in 61 patients (21.9%) and 49 patients (17.6%) died of disease. Table 3 presents a list regarding the use of ACEIs and ARBs. Even though patients receiving ACEIs or ARBs had higher incidences of cardiovascular disease and administration of other antihypertensive drugs, no significant difference was found between patient age, gender, the presence of CKD and diabetes, status of perioperative chemotherapy, tumour location, tumour length, and pathological features in surgical specimens. Using Kaplan–Meier analysis, we estimated the metastasis-free and disease-specific survival rates in patients administered or not administered ACEIs and ARBs (Figure 1A and B). The 5-year metastasis-free survival rate was 93.0% in patients administered ACEIs and ARBs and 72.8% in their counterparts (*P*=0.008), and the 5-year disease-specific survival rate was 93.2% in patients administered ACEIs and ARBs and 79.2% in their counterparts (*P*=0.028), respectively.

Univariate and multivariate analyses were performed to determine risk factors for subsequent tumour metastasis (Table 4). Univariate analysis revealed that patient age (*P*=0.048), no ACEI or ARB administration (*P*=0.013), the use of perioperative chemotherapy (*P*=0.009), tumour grade (*P*=0.001), pathological T stage (*P*<0.001), and LVI (*P*<0.001) were significant risk factors for predicting subsequent tumour metastasis. On multivariate analysis controlling these significant variables, no ACEI or ARB administration (*P*=0.027 HR=3.14) was an independent risk factor for a decrease in subsequent metastasis-free survival in addition to other prognostic indicators such as the appearance of pathological T3 (*P*=0.001, HR=3.50) and positive LVI (*P*=0.013, HR=2.10) in surgical specimens.

Next, we performed univariate and multivariate analyses to determine risk factors for disease-specific survival (Table 4). Univariate analysis revealed that the presence of CKD (*P*=0.014), no ACEI or ARB administration (*P*=0.039), tumour grade (*P*=0.003), pathological T stage (*P*<0.001), and LVI (*P*<0.001) were significant risk factors for a decrease in subsequent disease-specific survival. On multivariate analysis controlling the presence of CKD, the use of ACEIs or ARBs, tumour grade, pathological T stage, and LVI, the appearance of pathological T3 (*P*=0.007, HR=3.28) and positive LVI (*P*=0.049, HR=1.96) were independent risk factors for a decrease in subsequent disease-specific survival, whereas the use of ACEIs or ARBs was not an independent predictor of subsequent disease-specific survival. In subgroup analysis using 153 patients who had pT3 tumours, multivariate analysis also showed that no ACEI or ARB administration was an independent risk factor for a decrease in subsequent metastasis-free survival, whereas it did not independently predict disease-specific survival.

**Figure 2:**
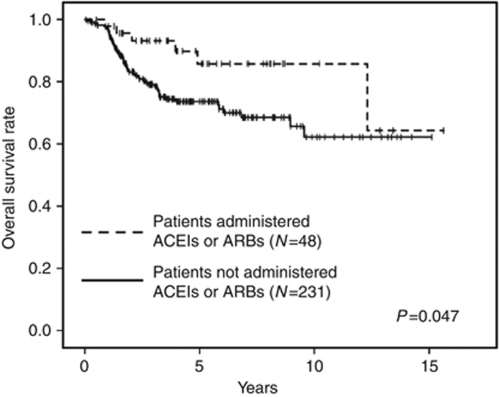
Overall survival rate after nephroureterectomy in patients administered *vs* not administered ACEIs or ARBs.

We then evaluated the associations between ACEI or ARB use and overall survival. A total of 62 subjects died during follow-up. Using Kaplan–Meier analysis, the 5-year overall survival rate was 85.8% in patients administered ACEIs and ARBs and 73.7% in their counterparts (*P*=0.047) (Figure 2), whereas multivariate analysis showed that ACEI or ARB use did not independently predict subsequent overall survival in our population.

## Discussion

In the present study, we retrospectively investigated the use of RAS inhibitors (ACEIs and ARBs) and other standard prognostic factors in 279 patients who underwent nephroureterectomy for localised UTUC. Kaplan–Meier analysis revealed that patients administered ACEIs or ARBs had significantly suppressed subsequent tumour metastasis. Multivariate analysis showed that in addition to other standard prognostic factors, ACEI or ARB use was an independent predictor of a decrease in metastasis-free survival, although it did not independently predict disease-specific survival. These results suggest that RAS inhibitor administration may improve disease control by suppressing subsequent tumour metastasis following radical surgery in patients with localised UTUC.

RAS inhibitors, such as ACEIs and ARBs, are widely used to treat hypertension, and there has been an increase in the number of reports evaluating the importance of RAS inhibition in organ protection, such as in the treatment of cardiac hypertrophy, diabetic nephropathy, and diabetic retinopathy (Grandi and Maresca, 2006). With respect to anti-tumourgenesis, Lever *et al* in a large retrospective cohort consisting of 5207 patients reported the first clinical evidence that long-term use of ACEs induced potent protective effects against carcinogenesis, whereas no significant association was apparent with the use of other antihypertensive drugs (Lever *et al*, 1998). Since then, in addition to cardiovascular functions regulated by the systemic RAS, the potential role of local RAS in malignancy has been recognised (Egami *et al*, 2003; Juillerat-Jeanneret *et al*, 2004; Ager *et al*, 2008; George *et al*, 2010), and an increasing body of evidence in experimental studies suggests that Ang II could act on tumour progression via two main and different mechanisms; the promotion of cancer proliferation and/or neovascularisation through AT1R (Greco *et al*, 2003; Arrieta *et al*, 2005; Khakoo *et al*, 2008).

Several researchers have investigated the clinical impact of RAS inhibitors in the treatment of malignancy. With respect to carcinogenesis, Ronquist *et al* reported that users of ACEI captopril had a lower risk of developing prostate cancer (Ronquist *et al*, 2004). Christian *et al* conducted a randomised prospective study using 1051 patients at high risk of keratinocyte cancer, and reported that users of ACEIs or ARBs had statistically significant reduced risks of basal cell carcinoma and squamous cell carcinoma (Christian *et al*, 2008). However, some studies have reported no significant correlation between RAS inhibitor use and carcinogenesis in breast cancer and prostate cancer patients (Li *et al*, 2003; Perron *et al*, 2004; Fryzek *et al*, 2006). Sipahi *et al* performed meta-analysis and reported that patients receiving ARBs had a slightly but significantly higher risk of developing lung cancer (1.2%), although no significant correlation was found in other solid cancers (Sipahi *et al*, 2010). Two retrospective studies also suggested the potent risk of carcinogenesis in RAS inhibitor administration (Chang *et al*, 2011; Opelz and Döhler, 2011). Therefore, determining the exact role of RAS inhibitors in carcinogenesis is controversial at the present time because of the existence of apparently contradictory evidence.

On the other hand, with respect to cancer treatment, a few reports evaluated the efficacy of RAS inhibitor administration, and most reported positive outcomes, especially in combination with other anti-cancer agents (Uemura *et al*, 2005; Wilop *et al*, 2009; Nakai *et al*, 2010; Tatokoro *et al*, 2011). Wilop *et al* analysed retrospectively 287 patients with advanced non-small cell lung cancer undergoing first-line platinum-based chemotherapy, and reported that patients receiving either ACEIs or ARBs had a median survival that was 3.1 months longer than non-recipients (11.7 *vs* 8.6 months) (Wilop *et al*, 2009). Nakai *et al* reported the use of ACEIs or ARBs with gemcitabine was an independent prognostic factor for both progression-free survival and overall survival in 155 patients with advanced pancreatic cancer (Nakai *et al*, 2010). Two prospective studies suggested RAS inhibitor administration was effective as a salvage therapy in the treatment of prostate cancer and renal cell cancer (Uemura *et al*, 2005; Tatokoro *et al*, 2011).

However, few studies have evaluated the possible role of RAS inhibitors as an adjuvant treatment following initial therapy. Yoshiji *et al* presented clinical evidence demonstrating that inhibition of RAS contributed to the suppression of tumour recurrence (Yoshiji *et al*, 2009). They demonstrated that ACEIs in combination with vitamin K suppressed the recurrence of hepatocellular carcinoma after curative therapy, although the exact impact of a single ACEI agent by itself could not be fully evaluated due to the limited number of patients in the study.

For patients with UTUC, local recurrence following radical surgery is rare and the risk of distant metastasis is directly related to subsequent prognosis (Rouprêt *et al*, 2011). To improve prognostic outcomes in high-risk UTUC, perioperative chemotherapy has been considered in both neoadjuvant and adjuvant settings (Hellenthal *et al*, 2009; Matin *et al*, 2010). However, limitations of a chemotherapeutic approach include the probability that some patients may not tolerate both chemotherapy and surgery (Kaag *et al*, 2010; Lane *et al*, 2010). Poor renal function following nephroureterectomy may also preclude the effective dose and/or administration of chemotherapeutic agents (Kaag *et al*, 2010). Thus, a new strategy that is both effective and possesses adequate tolerability would be a very important breakthrough in the adjuvant setting following nephroureterectomy. The present study may provide a potent aspect of RAS inhibitors as an effective treatment in UTUC.

Although tumour metastasis is affected by many factors, it may be controversial as to whether RAS regulation is a key mechanism involved in suppressing tumour metastasis. Our recent work has found that Ang II-AT1R signalling can have an effect on the tumour microenvironment by promoting macrophage mobilisation and infiltration into the tumour bed via signalling pathways involving monocyte chemoattractant protein-1 (MCP-1) (Shirotake *et al*, 2011). MCP-1 has been identified as a prominent regulator of the growth, survival, invasiveness, and migration of tumour cells (Egami *et al*, 2003; Li *et al*, 2009). Therefore, we believe that the regulation of RAS may potentially function to suppress subsequent tumour metastasis following surgery. However, in the present study, the use of RAS inhibitors could not independently predict disease-specific survival. We propose that one of the reasons might be the lack of CDDP-based chemotherapy after tumour metastasis. Focusing on patients who had tumour metastasis in our population, although half of the patients in the RAS inhibitor group did not receive CDDP-based regimens, 64.9% of the patients not administered RAS inhibitors were administered CDDP-based chemotherapy after tumour metastasis. This could be one of limitations in the study.

There is also another limitation. First, it was performed in a retrospective manner, and unknown sources of bias may exist in the findings. Due to the limited sample size of patients with ACEIs or ARBs, we could not fully evaluate the differences in doses or types of ACEIs and ARBs. Although the other antihypertensive drugs used consisted mainly of calcium-channel blockers and *β*-blockers or diuretics administered in a few patients, we did not fully evaluate the exact impact of *β*-blockers and diuretics on survival. We did not determine accurate compliance rates with respect to their medication or validate their compliance because our data were obtained using the information from patient interviews. In addition, we did not fully evaluate the pre-operative duration or past history of RAS inhibitor administration, although the hypertension in most of the patients administered RAS inhibitors had already been treated for a mean of 8.6 years duration pre-operatively. Not all patients received neoadjuvant and adjuvant chemotherapy, which may have had an effect on subsequent tumour progression. Thus, a prospective study with a larger population would be warranted in order to clarify the accurate prognostic role of RAS inhibitors in the treatment of UTUC.

In summary, the results of our retrospective analysis suggest that RAS inhibitor administration may improve prognostic outcomes in patients with UTUC. As RAS inhibitors are already used as antihypertensive drugs without severe side effects, we propose from a clinical point of view that they may be an effective choice in the treatment of patients with localised UTUC following nephroureterectomy.

## Figures and Tables

**Table 1:** Clinicopathological parameters in the 279 study patients

**Characteristic**	**No. of patients (%)**
*Age*	
<70 years	130 (46.6)
⩾70 years	149 (53.4)
	
*Gender*	
Male	207 (74.2)
Female	72 (25.8)
	
*Cardiovascular disease*	
Yes	34 (12.2)
No	245 (87.8)
	
*Chronic kidney disease*	
Yes	51 (18.3)
No	228 (81.7)
	
*Diabetes*	
Yes	36 (12.9)
No	243 (87.1)
	
*Hypertension*	
Yes	128 (45.9)
No	151 (54.1)
	
*Perioperative chemotherapy*	
Yes	45 (16.1)
No	234 (83.9)
	
*Tumour location*	
Renal pelvis	156 (55.9)
Ureter	123 (44.1)
	
*Tumour length*	
<30 mm	173 (62.0)
⩾30 mm	106 (38.0)
	
*Tumour grade*	
G1/2	108 (38.7)
G3	171 (61.3)
	
*Pathological T stage*	
<pT3	126 (45.2)
⩾pT3	153 (54.8)
	
*Lymphovascular invasion*	
Negative	170 (60.9)
Positive	109 (39.1)
	
*Concomitant CIS*	
Negative	223 (79.9)
Positive	56 (20.1)

Abbreviation: CIS=carcinoma *in situ*.

**Table 2:** Number of patients receiving antihypertensive drugs

**Characteristic**	**No. of patients**
*ACEIs*	5
Enalapril	3
Cilazapril	1
Perindopril	1
	
*ARBs*	43
Valsartan	16
Candesartan	15
Losartan	5
Telmisartan	4
Olmesartan	3
	
Calcium-channel blockers	90
*β*-blockers	15
Diuretics	7

Abbreviations: ACEIs=angiotensin-converting enzyme inhibitors; ARBs=angiotensin II receptor blockers.

**Table 3:** Clinicopathological parameters in 279 patients according to ACEI or ARB administration

**Characteristic**	**Patients not administered ACEI/ARB (%)**	**Patients administered ACEI/ARB (%)**	***P*-value**
No. of patients	231	48	
			
*Age*			0.470
<70 years	107 (46.3)	23 (47.9)	
⩾70 years	124 (53.7)	25 (52.1)	
			
*Gender*			0.476
Male	172 (74.5)	35 (72.9)	
Female	59 (25.5)	13 (27.1)	
			
*Cardiovascular disease*			0.016
Yes	23 (10.0)	11 (22.9)	
No	208 (90.0)	37 (77.1)	
			
*Chronic kidney disease*			0.533
Yes	42 (18.2)	9 (18.8)	
No	189 (81.8)	39 (81.2)	
			
*Diabetes*			0.138
Yes	27 (11.7)	9 (18.8)	
No	204 (88.3)	39 (81.2)	
			
*Use of non-ACEI/ARB drugs*			<0.001
Yes	65 (28.1)	34 (70.8)	
No	166 (71.9)	14 (29.2)	
			
*Perioperative chemotherapy*			0.529
Yes	37 (16.0)	8 (16.7)	
No	194 (84.0)	40 (83.3)	
			
*Tumour location*			0.333
Renal pelvis	131 (56.7)	25 (52.1)	
Ureter	100 (43.3)	23 (47.9)	
			
*Tumour length*			0.110
<30 mm	139 (60.2)	34 (70.8)	
⩾30 mm	92 (39.8)	14 (29.2)	
			
*Tumour grade*			0.102
G1/2	85 (36.8)	23 (47.9)	
G3	146 (63.2)	25 (52.1)	
			
*Pathological T stage*			0.112
<pT3	100 (43.3)	26 (54.2)	
⩾pT3	131 (56.7)	22 (45.8)	
			
*Lymphovascular invasion*			0.145
Negative	137 (59.3)	33 (68.7)	
Positive	94 (40.7)	15 (31.3)	
			
*Concomitant CIS*			0.335
Negative	183 (79.2)	40 (83.3)	
Positive	48 (20.8)	8 (16.7)	
			
No. of tumour metastasis	57 (24.7)	4 (8.3)	0.008
No. of cancer death	46 (19.9)	3 (6.4)	0.026

Abbreviations: ACEI=angiotensin-converting enzyme inhibitor; ARB=angiotensin II receptor blocker; CIS=carcinoma *in situ*.

**Table 4:** Risk factors for predicting metastasis-free and disease-specific survival following nephroureterectomy in 279 patients

	**Metastasis-free survival**			**Disease-specific survival**		
	**Univariate**	**Multivariate**		**Univariate**	**Multivariate**	
**Characteristic**	***P*-value**	**HR (95%CI)**	***P*-value**	***P*-value**	**HR (95% CI)**	***P*-value**
Age (<70 years *vs* ⩾70 years)	0.048			0.197		
Gender (male *vs* female)	0.105			0.249		
Cardiovascular disease (yes *vs* no)	0.956			0.449		
Chronic kidney disease (yes *vs* no)	0.126			0.014		
Diabetes (yes *vs* no)	0.129			0.922		
Hypertension (yes *vs* no)	0.553			0.874		
Use of non-ACEI/ARB drugs (yes *vs* no)	0.896			0.703		
Use of ACEI/ARB drugs (yes *vs* no)	0.013	3.14 (1.14–8.67)	0.027	0.039		
Perioperative chemotherapy (yes *vs* no)	0.009			0.122		
Tumour location (renal pelvis *vs* ureter)	0.124			0.575		
Tumour length (<30 *vs* ⩾30 mm)	0.060			0.165		
Tumour grade (G1/2 *vs* G3)	0.001			0.003		
Pathological T stage (<pT3 *vs* ⩾pT3)	<0.001	3.50 (1.63–7.49)	0.001	<0.001	3.28 (1.39–7.74)	0.007
Lymphovascular invasion (negative *vs* positive)	<0.001	2.10 (1.17–3.79)	0.013	<0.001	1.96 (0.99–3.97)	0.049
Concomitant CIS (negative *vs* positive)	0.786			0.559		

Abbreviations: ACEI=angiotensin-converting enzyme inhibitor; ARB=angiotensin II receptor blocker; CI=confidence interval; CIS=carcinoma *in situ*; HR=Hazard ratio.
